# Why is Housing Always Satisfactory? A Study into the Impact of Preference and Experience on Housing Appreciation

**DOI:** 10.1007/s11205-012-0114-9

**Published:** 2012-06-29

**Authors:** Sylvia J. T. Jansen

**Affiliations:** OTB Research Institute for the Built Environment, Delft University of Technology, PO Box 5030, 9600 GA Delft, The Netherlands

**Keywords:** Preference, Housing, Experience, Satisfaction

## Abstract

This study focuses on residents’ perceptions of residential quality concerning 23 different dwelling aspects. Respondents were asked to indicate their appreciation of these dwelling aspects on a scale ranging from 0 (“extremely unattractive”) to 100 (“extremely attractive”). The influence of two potential factors on the appreciation of dwelling aspects is examined: (1) preference and (2) experience. It was hypothesized that residents who live according to their preferences give higher appreciation scores than residents who do not. This should even apply to low-quality housing. Furthermore, it was argued that residents appreciate their current housing situation more than residents who do not live in that particular housing situation. This effect should be independent of preference. The impact of both preference and of experience could be confirmed. The results also showed an interaction effect between preference and experience: the positive effect of experience on appreciation is larger in residents who live in a housing situation that they do not prefer. This result would be expected if the impact of experience works to decrease the ‘gap’ in residential satisfaction due to the discrepancy between what residents have and what they want. In conclusion, why is housing always satisfactory? In this paper, housing is satisfactory because the ‘gap’ between what residents want and what they have is small; residents seem to have realistic aspirations. Furthermore, residents appreciate what they already have, even if this is not what they prefer.

## Introduction

For many people, the ideal dwelling would be a spacious, detached house with a front door located close to urban facilities and a backyard located in a green and quiet environment, such as a public park. However, in practice, the ideal dwelling is not achievable for most people. Instead, people search for a dwelling that provides the greatest possible amount of residential satisfaction, given the standard constraints, such as budget and housing supply.

Residential satisfaction refers to individuals’ appraisal of the conditions of their residential environment in relation to their needs, expectations and achievements (Amérigo and Aragonés 1997). This implies that residential aspirations or preferences have a large influence on residential satisfaction. If the current housing situation is about similar to the aspirations, then satisfaction should occur (Galster and Hesser 1981; Galster 1985). If there is a discrepancy between the actual housing situation and the preferred housing situation, dissatisfaction may be present (Mason and Faulkenberry 1978; Gärling and Friman 2002; Amérigo 2002). This discrepancy is also known as the have-want discrepancy (e.g., Wu 2008). Thus, residential satisfaction provides an indication of the difference between a household’s actual and preferred housing situation (Galster and Hesser 1981).

Interestingly, however, research has shown that people can be satisfied even when housing conditions are poor (e.g., Fine-Davis and Davis 1982; St. John and Clark 1984; Amérigo and Aragonés 1990). This implies that residents seem to be able to reduce the dissatisfaction arising from any gap between the current living conditions and preferred living conditions. In housing, there are various ways to reduce such a gap, for example, by moving house, adjusting the dwelling (e.g., renovation) or by adapting one’s own ideas (Brown and Moore 1970; Michelson 1977; Galster and Hesser 1981; Galster 1985; Priemus 1986; Floor et al.^1996^; Permentier et al. 2011). The first two options are limited by constraints, such as financial resources and the pressure in the housing market. The mechanism underlying the third possibility is not quite understood. It is often seen as a process of adaptation, which may be caused by various factors.

One of these factors involves lowered aspirations. Amos et al. (1982) found that residents with fewer goods and services were equally or even more satisfied in almost every area of life than residents with more goods and services were. The researchers attributed this to a difference in the level of aspirations between the groups. A person with lower aspirations is likely to be satisfied with less. Similarly, St. John and Clark (1984) argue that some groups of people may have a lower evaluative standard with regard to residential satisfaction than other groups have. When it comes to housing, this means that a relatively high satisfaction score for relatively poor housing quality could be explained by individual preferences.

Secondly, some researchers argue that people prefer what they already know or that they are satisfied with what they already have. For example, Kersloot and Kauko (2004) argue that tenants prefer housing types that they know from experience. Similarly, Priemus (1984) explains that the appreciation of certain aspects of housing will be higher if these aspects are part of the residents’ current housing situation. Priemus argues that people want what they know from their own housing situation and this means a confirmation of the status quo. Residents may have a tendency to evaluate the aspects of their current dwelling relatively positively because they are used to it. Thus, the finding that residents are reasonably satisfied even with relatively poor housing quality could be explained by the fact that residents are used to it.

In summary, residents who live in a suboptimal housing situation might reasonably be expected to experience a certain level of dissatisfaction. However, there are two factors that might reduce the level of dissatisfaction: (1) low aspirations or preferences and (2) actual experience. The first acts by lowering the aspirations or preferences and the second one by increasing the appreciation of the actually experienced housing situation. This paper explores the impact of these two factors on residents’ perceptions of residential quality concerning different aspects of their dwelling. As Priemus (1986) argues, in order to gain insight into people’s residential situation, it is of great importance to analyze the adaptation mechanisms that have occurred and continue to occur.

Knowledge of housing satisfaction is important because housing is the single most important item of consumption, with households spending approximately 25 % of their income on buying or renting a dwelling (Clark and Dieleman 1996; Dieleman 1996). Furthermore, housing also provides security, privacy, a neighborhood and social relationships, status, community facilities and services, access to jobs and control over the environment (Vera-Toscano and Ateca-Amestoy 2008). Housing is therefore an important component of individual well-being and quality of life (Vera-Toscano and Ateca-Amestoy 2008). Knowledge of the determinants of housing satisfaction can be used to design more effective housing programs and prevent problems arising from mismatches between the perceptions of policy makers and residents (Weidemann et al. 1982; Lu 1999; Vera-Toscano and Ateca-Amestoy 2008). Furthermore, this knowledge is also critical for a better understanding of decision processes underlying household mobility (Lu 1999).

Based on the considerations described above, the following alternative hypotheses have been formulated:

1. The impact of preference: Residents who live in accordance to their preferences have higher appreciation scores than residents who do not. This should even apply to low-quality housing;
2. The impact of experience: Residents appreciate their current housing situation better than residents who do not live in that particular housing situation. This effect should be independent of preferences.

## Methods

### Study Design and Respondents

The data for the study presented in this paper was collected as part of the large study “House Buyers in Profile” (in Dutch: “Huizenkopers in Profiel”; Boumeester et al. 2008a), which has been performed every 1 or 2 years in the Netherlands since 1995. In this study, data on residential preferences and the current housing situation was collected from residents on a standard income or higher; this applies to approximately 72 % of all Dutch households. The goal of the “House Buyers in Profile” study was to determine the needs and wishes of future homebuyers in order to establish what type of housing should be built. The questionnaire could only be answered by homeowners, tenants or their partners.

The data for the current study was collected though telephone interviews in the spring of 2008. A sample consisting of 6,169 addresses was obtained from a marketing bureau. After sending an introductory letter, 5,579 potential respondents were approached to participate in the study. The remaining 590 addresses were not contacted because we had collected enough telephone interviews. Of the 5,579 potential respondents, 3,000 (54 %) agreed to take part and 1,558 (28 %) refused. The remainder could not be contacted within the interview schedule (result last dial: no answer (n = 439), busy (n = 19), appointment (n = 59), answering machine (n = 84), disconnected (n = 221), and other (n = 199); total n = 1,021; 18 %). The respondents were stratified according to region (north, east, south and west) so that the final sample contained approximately 25 % from each region. This aim was achieved (North: n = 669 (22 %), East: n = 776 (26 %), South: n = 760 (25 %), West: n = 795 (26 %).

One goal of the House Buyers in Profile study was to explore the housing preferences of respondents who were willing to move. As such, an important question during the telephone interview was whether respondents would be willing to move if they found a dwelling that would meet all their housing needs. Note that the exact formulation of this question is presented in the Appendix together with other relevant questions. About one-third of the respondents (n = 1,074; 36 %) indicated that they were willing to move in such a situation while 1,907 respondents were unwilling to move (willingness to move was unknown for 19 of the respondents). Due to budgetary constraints, about half the respondents in the latter group (n = 886; 46 %) were presented with a very short version of the survey in which no socio-demographic characteristics were obtained. For the other half (n = 1,021; 54 %) as well as for the respondents who were willing to move (n = 1,074), information on socio-demographic characteristics was collected.

The socio-demographic characteristics of both groups are provided in Table 1. For reasons of comparison, an overview of the characteristics of the upper 70 % of households in the Netherlands in 2008 with regard to income is also provided (source: Central Bureau for Statistics Netherlands (CBS) http://statline.cbs.nl). The upper 70 % of households with regard to income was chosen because the respondents in the current study were selected as having at least a standard income; this applies to about 72 % of all Dutch households. Note, however, that in practice the information on income was not always up-to-date and 17 % of the respondents (n = 302 of 1,772 respondents with information on income) turned out to have a lower than standard monthly net income (2008: € 1,768). When compared to the upper 70 % of households with regard to income, the present sample included a relatively high number of respondents aged between 45 and 64 years of age. The sample seems reasonably representative in terms of the other characteristics. The hypotheses regarding the impact of preference and experience are explored in the group of respondents who is willing to move because this group is supposed to perceive dissatisfaction due to the discrepancy between what they have and what they want.

**Table 1:** Socio-demographic characteristics

	Respondents who are not willing to move	Respondents who are willing to move (sample used in current paper)	The upper 70 % of households in the Dutch population in 2008 with regard to income
Age	n = 1,014	n = 1,032	n = 5,070,000
<25 years	10 (1 %)	8 (1 %)	64,000 (1 %)
25–44 years	226 (22 %)	340 (33 %)	1,891,000 (37 %)
45–64 years	583 (57 %)	552 (53 %)	2,139,000 (42 %)
65 and older	195 (19 %)	132 (13 %)	976,000 (19 %)
Household type	n = 1,015	n = 1,032	n = 5,072,000
Single	164 (16 %)	133 (13 %)	840,000 (17 %)
Couple without children <18	445 (44 %)	393 (38 %)	1,859,000 (37 %)
Couple with children <18	372 (37 %)	448 (43 %)	1,860,000 (37 %)
Other	34 (3 %)	58 (6 %)	513,000 (10 %)
Number of persons in the household	n = 1,015	n = 1,032	n = 5,069,000
One	164 (16 %)	133 (13 %)	840,000 (17 %)
Two	466 (46 %)	431 (42 %)	2,075,000 (41 %)
Three	132 (13 %)	162 (16 %)	820,000 (16 %)
Four	171 (17 %)	201 (20 %)	928,000 (18 %)
Five or more	82 (8 %)	105 (10 %)	406,000 (8 %)
Monthly net income^a^	n = 875	n = 883	n = 5,068,000
Mean	2,701 (1,134)	2,693 (1,150)	3,455
Education	n = 982	n = 1,009	Unknown
Primary/lower vocational education	235 (24 %)	186 (18 %)	
Secondary education	341 (35 %)	383 (38 %)	
Higher vocational education	317 (32 %)	347 (34 %)	
University	58 (3 %)	67 (7 %)	
Other	31 (3 %)	26 (3 %)	
Gender	n = 1,012	n = 1,054	Not applicable
Female	553 (55 %)	550 (52 %)	
Paid work (n = 1,031)	n = 1,014	n = 1,031	Unknown
Yes	623 (61 %)	702 (68 %)	

^a^Seven respondents with a standardized score >5 (i.e., a monthly net income >€ 10.000) were omitted from the analyses because they are extreme outliers

### The Set of Dwelling Aspects

The set of dwelling aspects included in this study is based on the literature and on previous studies (Floor and van Kempen 1994; Goetgeluk 1997; Heins 2002; Boumeester et al. 2005, 2008b; Jansen et al. 2009). We included seven aspects that pertained to the dwelling and one that pertained to the dwelling environment. The dwelling aspects and their levels are presented in Table 2.

**Table 2:** Dwelling aspects and levels

Categorical dwelling aspects	Numerical dwelling aspects
Dwelling type	Purchase costs/Rental costs
Apartment	€ 140,000/€ 338 per month
Terraced house/corner house	€ 220,000/€ 532 per month
Semi-detached house	€ 300,000/€ 725 per month
Tenure	Size of the living room (m^2^)
Rental house	20
Owner-occupied house	30
	40
Architectural style	Number of rooms
Traditional	2
Modern	3
Innovative	4
Residential environment	Size of backyard/balcony (m/m^2^)
Urban	5/4
Sub-urban	10/7
Rural	15/10

Respondents were asked about their actual housing situation with regard to the eight aspects. They were therefore asked which type of dwelling they occupied, the availability and size of a garden or balcony, the architectural style of their dwelling, and so on. For the numerical dwelling aspects, the responses were classified into categories. For example, the actual size of the living room was recoded into four categories: <25, 25–34, 35–44 and more than 44 m^2^. Next, respondents with a living room of <25 m^2^ were classified as having a living room of 20 m^2^, respondents with a living room between 25 and 34 m^2^ were classified as having a living room of 30 m^2^ and respondents with a living room between 35 and 44 m^2^ were classified as having a room of 40 m^2^. This was done in order to be able to compare the mean appreciation scores between respondents living in the particular housing situation and those not living in the particular housing situation (and, thus, the impact of experience). Note that the aspect of cost was not analyzed because remaining mortgage and the perceived actual value of the dwelling had been asked about, but not the purchase price.

Aside from their current housing situation, the respondents were also asked about their preferences with regard to the eight dwelling aspects. Thus, they were asked which type of dwelling they preferred, the preferred architectural style of their dwelling, and so on. For the numerical dwelling aspects, the responses were classified into categories in the same way as described for the actual dwelling characteristics.

### The Appreciation Scores

We obtained the respondents’ appreciation scores for the different dwelling aspects shown in Table 2 with the use of rating scales. Respondents were asked to indicate their appreciation of each level of every dwelling aspect on a scale with two anchors: “extremely unattractive” (with an assigned value of 0) and “extremely attractive” (with a value of 100). The questions were introduced by explaining these endpoints and by stating that the higher the number awarded, the more attractive that aspect was. Furthermore, the interviewer explained that the respondent had to take his/her actual housing situation and household income as a starting point when answering the questions.

The respondents rated all the dwelling aspects described in Table 2, irrespective of whether or not they actually lived in that particular housing situation. However, the questions with regard to the three aspects of length of the backyard and of size of the balcony were asked only to respondents with a backyard or balcony, respectively.

## Results

Respondents gave appreciation scores for 23 dwelling aspects, including aspects of their own current housing situation. The mean appreciation scores for the respondents’ current housing situation are shown in Table 3. For example, 172 respondents were currently living in an apartment. Seventy of these respondents (41 %) were not willing to move. It was assumed that they were living in an optimal housing situation; they gave a mean appreciation score of 85.7 for an apartment. By contrast, 102 respondents were living in an apartment but would move if they found another dwelling that fulfilled all their housing needs. It was assumed that these residents were living in a suboptimal housing situation; they gave a mean appreciation score of 71.8 for an apartment.

**Table 3:** Mean appreciation scores for various aspects of the current housing situation in respondents who are willing to move and those who are not

	Not willing to move (optimal housing situation)		Willing to move (suboptimal housing situation)		Statistically significant covariates (*p* ≤ 0.05)	*p* value difference between groups
	Mean	n	Mean	n		
Dwelling type						
Apartment	85.7	70	71.8	102	–	*p* < 0.01
Terraced/corner house	78.2	403	67.7	464	Age, number of persons, gender	*p* < 0.01
Semi-detached house	78.6	176	71.0	180	Age, number of persons, income, paid work	*p* < 0.01
Tenure						
Rental house	82.6	181	71.0	271	Age	*p* < 0.01
Owner-occupied house	88.8	698	84.0	626	Age, income	*p* < 0.01
Architectural style						
Traditional	80.1	794	79.9	768	Number of persons, gender	*p* = 0.69
Modern	69.1	146	68.3	176	–	*p* = 0.76
Innovative	77.4	41	70.0	44	Gender, paid work	*p* = 0.07
Residential environment						
Urban	61.3	117	60.3	130	Number of persons	*p* = 0.77
Sub-urban	63.5	365	62.0	409	Age	*p* = 0.22
Rural	80.7	520	78.6	474	Number of persons	*p* = 0.09
Size living room						
20 m^2^/<25 m^2^	57.8	89	41.1	124	Age	*p* < 0.01
30 m^2^/25–34 m^2^	68.6	216	63.2	271	Income	*p* = 0.01
40 m^2^/35–44 m^2^	78.7	300	76.7	268	–	*p* = 0.27
Number of rooms						
2/1–2 rooms^a^	75.0	20	41.3	30	–	–
3/3 rooms	75.1	89	62.5	106	Number of persons	*p* < 0.01
4/4 rooms	75.1	269	73.8	292	Number of persons	*p* = 0.93
Backyard size						
5 m/<8	54.6	102	47.1	97	–	*p* = 0.04
10 m/8–12 m	67.2	343	59.3	377	–	*p* < 0.01
15 m/13–17 m	73.3	139	67.1	177	Age	*p* = 0.01
Size balcony						
4 m^2^/<6 m^2a^	51.8	19	44.6	48	–	–
7 m^2^/6–8 m^2a^	67.6	23	52.6	24	–	–
10 m^2^/9–12 m^2a^	73.6	18	59.0	24	–	–

^a^Not analyzed statistically

Table 3 shows that in both groups the highest appreciation score was given for an owner-occupied house. A living room of 20 m^2^ and a dwelling with two rooms was rated with the lowest mean appreciation scores in respondents living in a suboptimal housing situation. In the optimal housing situation, the lowest mean appreciation score was given for a balcony of 4 m^2^.

The first analysis investigates whether residents who live in a suboptimal housing situation show a lower level of residential satisfaction than respondents who live in an optimal housing situation. In general, the latter respondents gave higher mean appreciation scores for the dwelling aspects than respondents living in a suboptimal housing situation. This was tested statistically using univariate analysis of variance. In each of the analyses, the appreciation score for a particular housing aspect (e.g., an apartment) was included as the dependent variable. The willingness to move was included as an independent variable (fixed factor), separating residents living in an optimal housing situation from those living in a suboptimal situation. Age, income, gender, number of persons in the household and engaged in paid work were included as covariates in the analyses in order to correct for the impact of differences in the socio-demographic characteristics of the two groups. For example, higher age, higher income and female gender were related to higher residential satisfaction in the study by Perez et al. (2001). By including these characteristics in the analyses, the comparison of the appreciation scores between the various groups was not influenced by them. Covariates are only retained in the ultimate models if they showed a statistically significant influence (*p* < 0.05). Due to the low number of cases (<30 in either one of the groups), four dwelling aspects were not analyzed statistically: two rooms and all aspects of the size of the balcony.

The socio-demographic characteristics were found to be related to the appreciation scores in the following way. Higher age was statistically significantly (*p* ≤ 0.05) related to lower appreciation of a terraced/corner house, a semi-detached house, an owner-occupied house, a suburban residential environment, a living room of 20 m^2^ and a backyard of 15 m length. Higher age was also related to higher appreciation of a rental house. Women recorded higher appreciation of a terraced/corner house, a traditional architectural design and an innovative architectural design. A higher number of persons in the household was related to lower appreciation of an urban residential environment, a dwelling with three rooms and a dwelling with four rooms. A higher number of persons in the household was also related to higher appreciation of a terraced/corner house, a semi-detached house, a traditional architectural style and a rural residential environment. A higher income was related to lower appreciation of a semi-detached house and a living room of 30 m^2^ and to a higher appreciation of an owner-occupied house. Finally, engaged in paid work was positively related to appreciation of a semi-detached house and an innovative architectural design.

In total, statistically significant differences (*p* < 0.05) were observed for 11 of the 19 dwelling aspects analyzed (58 %), all of which pointed in the same direction: residents who live in a suboptimal housing situation have lower mean appreciation scores than residents who live in an optimal housing situation. This difference seems to reflect the dissatisfaction caused by a gap between the actual and preferred housing situation. The remaining dwelling aspects do not show a statistically significant difference in the mean appreciation score between the two groups. This could indicate the functioning of a psychological mechanism, such as experience or preference, that reduces residential dissatisfaction. Furthermore, psychological mechanisms might have reduced residential dissatisfaction even though statistically significant differences remain. The impact of the psychological mechanisms is explored in the next section but only in respondents who experience a discrepancy (a ‘gap’) between what they have and what they want (those respondents who are willing to move). These are assumed to be experiencing dissatisfaction with their current housing situation to some degree.

### The Mean Appreciation Scores

The first hypothesis argues that residents who live in the way they would prefer have higher appreciation scores than residents who do not. This should even apply to a housing situation of relatively low quality, such as a dwelling with two rooms, a living room of 20 m^2^ and a backyard with a length of 5 m. The hypothesis regarding the impact of experience states that residents appreciate their actual housing situation better than residents who do not live in that particular housing situation. This should apply irrespective of whether or not residents prefer the particular dwelling aspect.

To explore these hypotheses, for every dwelling aspect the respondents were divided into those who lived in that particular housing situation (e.g., in an apartment) and those who do not (this measures ‘experience’ of that particular situation). Then, the respondents were also divided according to their preference for each of the dwelling aspects. In this way, four groups were formed for every dwelling aspect: (1) respondents who lived in the housing situation that they preferred (actual and preferred), (2) respondents who did not live in the housing situation that they preferred (not actual, preferred), (3) respondents who lived in a housing situation that was not their preferred situation (actual and not preferred), and, (4) respondents who did not live in the housing situation and would not want to do so either (not actual and not preferred). Both the second and third group show a discrepancy between what they have and what they want.

The mean appreciation scores for the various dwelling aspects are shown in Table 4. The Table can be read in the following way. There are 59 respondents who lived in an apartment and who also preferred living in an apartment (group 1). This group lived in accordance to their preference with regard to dwelling type. Their mean appreciation score for an apartment is 81.4. Group 2 does not currently live in an apartment but would like to do so (mean = 74.9). Twenty respondents currently lived in an apartment but would prefer to live in another type of dwelling (group 3); they had a mean appreciation score for an apartment of 38.2. Finally, 464 respondents did not live and did not want to live in an apartment; they had a mean appreciation score of 15.8 for an apartment.

**Table 4:** Mean appreciation scores for various aspects of the dwelling situation

	Actual housing situation, preferred (1)		Not actual housing situation, preferred (2)		Actual housing situation, not preferred (3)		Not actual housing situation, not preferred (4)	
	Mean	n	Mean	n	Mean	n	Mean	n
Dwelling type								
Apartment	81.4	59	74.9	309	38.2	20	15.8	464
Terraced/corner house	74.9	300	62.0	267	46.7	105	27.6	180
Semi-detached house	77.3	122	78.2	421	56.5	54	48.2	255
Tenure								
Rental house	80.5	170	67.3	166	49.3	74	21.9	450
Owner-occupied house	88.3	571	82.5	152	29.9	45	25.8	92
Architectural style								
Traditional	82.8	532	77.1	107	68.6	131	61.6	72
Modern	73.0	66	71.6	192	62.3	75	54.1	507
Innovative^a^	73.5	23	69.0	178	63.3	15	46.6	624
Residential environment								
Urban	67.8	59	56.1	114	52.9	47	39.9	640
Sub-urban	63.1	239	58.7	146	60.0	110	50.4	365
Rural	81.8	300	75.5	136	70.8	105	58.4	319
Size living room								
20 m^2^/<25 m^2a^	67.5	16	69.4	8	36.5	93	18.9	720
30 m^2^/25–34 m^2^	70.0	161	64.3	172	52.5	100	37.0	404
40 m^2^/35–44 m^2^	82.9	116	80.7	172	70.9	112	68.6	437
Number of rooms								
2/1–2 rooms^a^	80.1	1	75.0	6	40.4	27	12.3	828
3/3 rooms	82.1	36	75.0	182	48.6	51	28.1	593
4/4 rooms	81.9	103	79.6	200	69.2	154	55.1	405
Backyard size								
5 m/<8	60.4	33	60.7	45	34.3	38	22.5	453
10 m/8–12	72.9	158	71.2	88	51.5	104	41.7	219
15 m/13–17	84.3	41	78.5	72	69.0	76	63.6	380
Size balcony								
4 m^2^/<6 m^2a^	70.0	2	–	–	46.4	7	13.9	24
7 m^2^/6–8 m^2a^	76.7	3	40.0	2	29.0	3	36.8	25
10 m^2^/9–11 m^2a^	85.0	4	65.0	11	70.0	1	52.6	17

^a^Not analyzed statistically, due to low frequencies

In general, the mean appreciation scores exhibited the following trend; the highest mean appreciation score was found in the group of residents who live in their preferred housing situation. The next highest mean score is observed for the group of residents who preferred the particular housing situation but did not live there currently. The lowest mean score is generally found in the group who did not live in the particular housing situation and had no wish to do so either.

Table 4 shows that, generally, among respondents who lived in the particular housing situation, the mean appreciation scores of respondents who preferred this situation (group 1) were higher than the mean scores of respondents who did not want to live in this particular housing situation (group 3). Similarly, among the respondents who do not live in the particular housing situation, those who preferred that particular situation (group 2) indicated higher mean appreciation scores than those who did not (group 4). These results indicate that preference does have an impact on housing appreciation.

Furthermore, the mean appreciation scores of respondents who currently live in the housing situation that they prefer (group 1) were higher than the mean scores of respondents who do not currently live in the housing situation that they prefer (group 2). These two groups of respondents both prefer to live in the particular housing situation, but one group actually does (group 1) and the other group (group 2) does not. The first group generally had higher appreciation scores. This result points to the impact of experience. The same applies to groups 3 and 4. In both groups, respondents provide an appreciation score for a housing situation that they do not prefer. However, respondents who actually live in their non-preferred housing situation (group 3) generally had higher mean appreciation scores than respondents who do not live there (group 4).

Both hypotheses are tested statistically using analysis of variance. One by one, the appreciation scores for the dwelling aspects were included as dependent variables in the analyses. Group membership was included as a fixed factor in the analyses. The socio-demographic variables of age, income, gender, having paid work and number of persons in the household are included as covariates in the analyses. Six of the 23 dwelling aspects could not be tested statistically due to low frequencies: an innovative architectural design, a living room of 20 m^2^, a dwelling with 2 rooms and all levels of the size of the balcony.

The results of the statistical analyses are presented in Table 5. First, the results relating to the socio-demographic characteristics will be described. Age is statistically significantly (*p* < 0.05) related to nine of the seventeen dwelling aspects (53 %). Older respondents appreciate an apartment and a dwelling with three rooms more than younger respondents do. Furthermore, they have less appreciation of a terraced/corner house, a semi-detached house, an owner-occupied house, a living room of 30 and 40 m^2^, a backyard of 5 and of 15 m length than younger respondents. Income is statistically significantly related to ten dwelling aspects. Respondents with a higher income have a lower appreciation of a terraced/corner house, a semi-detached dwelling, a rental house, a modern architectural design, a living room of 30 and of 40 m^2^, a dwelling with 3 rooms and 4 rooms and a backyard of 5 and of 10 m length. The number of persons in the household is statistically significantly related to eleven dwelling aspects. A larger number of persons in the household is positively related to appreciation of an owner-occupied house, a rural residential environment and a living room of 40 m^2^. Furthermore, there is less appreciation for an apartment, an urban and suburban residential environment, a living room of 30 m^2^, a dwelling with three rooms, a dwelling with four rooms and a backyard of 5 and of 10 m. With regard to gender, women show a higher mean appreciation score for a traditional architectural design and a living room of 40 m^2^. Males have a higher mean appreciation for an urban residential environment. Finally, respondents engaged in paid work show a higher mean appreciation score for a terraced/corner house, a semi-detached house and a traditional architectural design when compared to respondents without paid work.

**Table 5:** Results of analyses of variance with the appreciation score for each particular dwelling aspect as dependent variable

Dwelling aspect evaluated/actual	Age	Income	Number of persons in household	Gender	Having paid work	Impact of preference	Impact of experience	Interaction preference and experience
Dwelling type								
Apartment (n = 852)	*p* < 0.01	No	*p* < 0.01	No	No	*p* < 0.01	*p* < 0.01	*p* < 0.01
Terraced/corner house (n = 852)	*p* < 0.05	*p* < 0.05	No	No	*p* < 0.05	*p* < 0.01	*p* < 0.01	No
Semi-detached house (n = 852)	*p* < 0.01	*p* < 0.05	No	No	*p* < 0.01	*p* < 0.01	*p* < 0.05	*p* < 0.05
Tenure								
Rental house (n = 860)	No	*p* < 0.05	No	No	No	*p* < 0.01	*p* < 0.01	*p* < 0.01
Owner-occupied house (n = 860)	*p* < 0.01	No	*p* < 0.05	No	No	*p* < 0.01	*p* < 0.01	No
Architectural style								
Traditional (n = 840)	No	No	No	*p* < 0.05	*p* < 0.01	*p* < 0.01	*p* < 0.01	No
Modern (n = 840)	No	*p* < 0.05	No	No	No	*p* < 0.01	*p* < 0.05	No
Innovative	–	–	–	–	–	–	–	–
Residential environment								
Urban (n = 860)	No	No	*p* < 0.01	*p* < 0.01	No	*p* < 0.01	*p* < 0.01	No
Sub-urban (n = 860)	No	No	*p* < 0.05	No	No	*p* < 0.01	*p* < 0.01	No
Rural (n = 860)	No	No	*p* < 0.01	No	No	*p* < 0.01	*p* < 0.01	*p* < 0.05
Size living room								
20 m^2^/<25	–	–	–	–	–	–	–	–
30 m^2^/25–34 m^2^ (n = 837)	*p* < 0.01	*p* < 0.01	*p* < 0.01	No	No	*p* < 0.01	*p* < 0.01	*p* < 0.05
40 m^2^/35–44 m^2^ (n = 837)	*p* < 0.05	*p* < 0.01	*p* < 0.05	*p* < 0.01	No	*p* < 0.01	No	No
Number of rooms								
2/1–2 rooms	–	–	–	–	–	–	–	–
3/3 rooms (n = 862)	*p* < 0.05	*p* < 0.05	*p* < 0.01	No	No	*p* < 0.01	*p* < 0.01	No
4/4 rooms (n = 862)	No	*p* < 0.01	*p* < 0.01	No	No	*p* < 0.01	*p* < 0.01	*p* < 0.05
Backyard size								
5 m/<8 (n = 569)	*p* < 0.05	*p* < 0.01	*p* < 0.05	No	No	*p* < 0.01	No	*p* = 0.08
10 m/8–12 (n = 569)	No	*p* = 0.05	*p* < 0.01	No	No	*p* < 0.01	*p* < 0.01	No
15 m/13–17 (n = 569)	*p* < 0.01	No	No	No	No	*p* < 0.01	No	No
Size balcony								
4 m^2^/<6 m^2^	–	–	–	–	–	–	–	–
7 m^2^/6–8 m^2^	–	–	–	–	–	–	–	–
10 m^2^/9–12 m^2^	–	–	–	–	–	–	–	–

### The Impact of Preference

The impact of preference is clear (see Table 5): respondents who prefer a particular dwelling aspect gave higher mean appreciation scores than respondents who do not prefer the particular dwelling aspect, irrespective of their own actual housing situation. Unfortunately, the effect could not be tested statistically in three dwelling aspects of relatively low housing quality (a living room of 20 m^2^, 2 rooms and a balcony of 4 m^2^) due to the low number of cases. The mean appreciation scores, however, show the same trend (see Table 4).

It is interesting to explore whether the impact of preference is so strong that relatively poor housing quality is appreciated as much as higher housing quality among respondents who live in the housing situation that they prefer. For example, are respondents who live in a dwelling with two rooms and who prefer this housing situation as appreciative of this dwelling aspect as respondents who live and prefer to live in a dwelling with four rooms? Analyses are performed in the same way as described above, thus with a correction for age, income, gender, paid work and number of persons in the household, but only for respondents who live in the housing situation that they prefer (the first column of Table 4). The analysis is performed only for the ‘numerical dwelling aspects’—that is, size of living room, number of rooms and backyard size. The results show that a living room of 40 m^2^ is more appreciated (n = 116: mean = 82.9) than a living room of either 30 m^2^ (n = 161: mean = 70.0) or 20 m^2^ (n = 16: mean = 67.5), but that there is no difference in mean appreciation between the latter two. With regard to the number of rooms, there is no difference in mean appreciation between respondents who live in a dwelling with three rooms (n = 36: mean = 82.1) and those who live in a dwelling with four rooms (n = 103: mean = 81.9). Appreciation for a dwelling with two rooms was not investigated because the number of cases was too low. Finally, respondents with a backyard of about 5 m length (n = 33: mean = 60.4) have a statistically significantly lower mean appreciation score compared to respondents with a backyard of 10 m in length (n = 158: mean = 72.9) and 15 m in length (n = 41: mean = 84.3). In addition, mean appreciation does differ statistically significantly between the latter two groups.

In conclusion, preference definitely impacts on residents’ appreciation of dwelling characteristics. In some cases, this may even lead to relatively poor housing quality being appreciated as much as higher quality housing.

### The Impact of Experience

The results presented in Table 5 show that experience has a statistically significant impact for 14 out of 17 of the dwelling aspects analyzed. This indicates that respondents who live in a particular housing situation show more appreciation of this situation than respondents who do not live in the particular situation, irrespective of preference. This means that there is evidence that experience of a particular housing situation increases one’s appreciation of that housing situation. The difference is especially notable between group 3 (respondents living in a housing situation that they do not prefer) and group 4 (respondents not living in the housing situation that they do not prefer). Thus, both groups do not prefer a particular housing situation, but the group (group 3) who lives in a particular housing situation generally has much higher appreciation scores for that situation (see Table 4).

### The Interaction between Preference and Experience

Interestingly, a statistically significant (*p* < 0.10) interaction effect is found for seven dwelling aspects. Note that the *p* value for an interaction effect to be termed statistically significant is set at *p* < 0.10, which is quite common for an interaction effect.

**Fig. 1:**
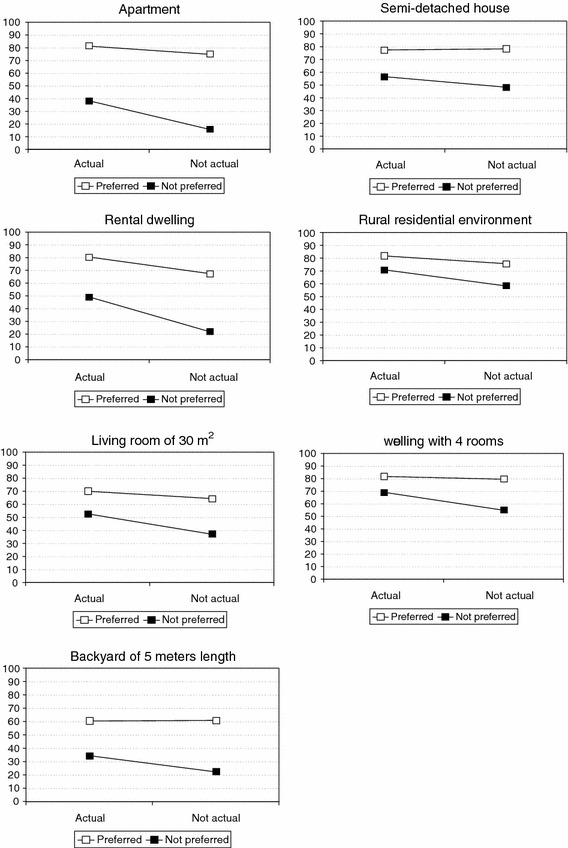
Dwelling aspects for which an interaction effect of preference and experience was observed

The dwelling aspects for which interaction effects were observed are shown in Fig. 1. The same mean appreciation scores as shown in Table 4 are now shown on line graphs in order to be able to interpret the interaction results more easily. The results show that for all seven dwelling aspects, the impact of experience is larger in the “not preferred” group than in the “preferred” group. Thus, the effect of respondents appreciating the housing that they have direct experience of is greater for dwelling aspects that they do not prefer than for dwelling aspects that they prefer. This result would be expected if the impact of experience works to decrease the ‘gap’ in residential satisfaction due to the discrepancy between what residents have and what they want.

## Discussion

This paper has explored the impact of two factors that might influence residents’ perceptions of residential quality concerning different aspects of their dwelling.

Firstly, the impact of preference was established. The first alternative hypothesis argued that residents who live in a housing situation that corresponds to their preferences will give higher mean appreciation scores than residents who live in a housing situation that they do not prefer. The results showed that the null-hypothesis of no difference could be rejected in favor of the alternative hypothesis. It was even shown that in some cases, dwelling aspects of lower quality were equally appreciated as dwelling aspects of higher quality. Marcuse (1971) pointed out that identical living conditions may have an entirely opposite influence on individual satisfaction. After all, one resident may live in a dwelling with only two rooms and be satisfied because of the easy upkeep while another may have the need for more privacy and therefore prefer a more spacious dwelling. Note, however, that the current study did not explore the underlying reasons for having particular preferences. If, for example, someone has a relatively low aspiration level because all his or her friends and relatives live in the same low-quality housing situation, then this would not be reflected in the current study. One of the factors that might have an impact on aspirations is social comparison. Amérigo and Aragonés (1997) explain that people may use a standard of residential quality that is determined by the subject’s group of reference and other factors. Christoph (2010) argues that the core idea of the relative standards model is that people may apply various standards in order to reach an opinion about their current [housing] situation, such as their previous [housing] situation or the current [housing] situation of important others in their lives. Hence, these authors refer to some kind of frame of reference that determines one’s aspirations or acts as a mediator between the evaluation of the objective aspects of the actual housing situation and satisfaction. Veenhoven (1996) poses (and disputes) the notion that people use relative standards of how life should be, which are drawn on perceptions on what is feasible and on comparisons with others. These standards change continuously: we may be satisfied when life comes close to ideal, but we then set ourselves higher goals and end up equally satisfied.

Secondly, the impact of experience was examined. It was hypothesized, on the basis of the literature (for example, Priemus 1984; Kersloot and Kauko 2004) that residents who actually live in a particular housing situation give higher appreciation scores for these aspects than residents not living in that particular housing situation, irrespective of whether or not they prefer this situation. Our results generally support this hypothesis. Furthermore, the impact of experience turned out to be stronger for non-preferred than for preferred dwelling aspects. This result strengthens the hypothesis. After all, residents who live in a housing situation that they are averse to need this mechanism to suppress negative feelings arising from the discrepancy between what they have and what they want, whereas residents who live in a housing situation that they prefer do not need this.

With regard to the impact of experience, it is interesting to discuss Helson’s (Helson 1947, 1964) Adaptation-Level theory. This theory argues that judgments about a stimulus are made relative to the context of this judgment, including previous experiences and peripheral stimuli (Russell and Lanius 1984). This means that judgments are adapted to the range and distribution of the available experiences (Russell and Lanius 1984 refer to Parducci 1968). Furthermore, Helson (1964) argues that adaptation levels appear as neutral or indifferent zones in bipolar responses. Thus, one judges a particular aspect on the basis of one’s own experience and the context of the evaluation task. As a result, persons take up a position on a bipolar scale on which they are indifferent (for example, three rooms is fine) and from this point of view two rooms might be viewed negatively and four rooms positively. In the current study, a particular effect of adaptation level is shown for the numerical dwelling aspects (size of the living room, number of rooms, length of the backyard and size of the balcony). Respondents who evaluated dwelling aspects with a lower housing quality than their current housing, gave lower appreciation scores for these dwelling aspects than respondents who evaluated dwelling aspects with a higher housing quality than their current housing. For example, respondents living in a dwelling with four rooms (n = 588) provided a mean appreciation score for a dwelling with three rooms of 43.9. By contrast, those living in a dwelling with 1 or 2 rooms (n = 50) provided a mean appreciation score of 69.8 for a dwelling with three rooms. Seen from the point of those with four rooms, a dwelling with three rooms might be seen as a ‘loss’ whereas residents living in a dwelling with 1 or 2 rooms might believe a dwelling of three rooms to be a ‘gain’. The same results were seen for the other levels of number of rooms as well as for the other numerical dwelling aspects (results not shown, table can be obtained from the researcher).

Apart from preference, experience, social comparison and an internal adaptation level, there are other factors that could influence residential satisfaction. Amérigo and Aragonés (1990) argue that the factors most relevant in explaining high levels of residential satisfaction despite suboptimal housing conditions are psychosocial factors, such as attachment to a particular place and social interactions or the networks that form between inhabitants. The impact of these factors was not examined in the current study. However, although such psychosocial factors may affect general satisfaction, it seems unlikely that they would play a role in the appreciation of various separate dwelling aspects (for example, a dwelling with two rooms), as explored in the current study. Furthermore, Veenhoven (1996) mentions the following factors that could explain relatively high general satisfaction scores: simple denial of one’s misery and a tendency to see things in a more positive light than the reality might imply. He also argues that people tend to enjoy their lives once conditions are tolerable. This means that residents living in really poor circumstances may give lower satisfaction scores but that above a certain level of comfort, people tend to be satisfied. Gärling and Friman (2002) describe the expectation-disconfirmation-performance (EDP) model of satisfaction by Oliver (1997). In short, performance (for example, low housing quality) could have a direct effect on satisfaction but the effect may also be indirect through disconfirmation (which is a trade-off between performance and expectations) or when combined with the effect of expectations.

Other potential mechanisms that may affect residential satisfaction are cognitive restructuring and future perspectives. Cognitive restructuring (sometimes also called cognitive dissonance reduction) is the tendency for individuals to seek consistency in their cognitive processes and states (e.g., their beliefs and opinions) or between cognitions and behavior. This is done in order to avoid negative feelings. If the actual dwelling situation is perceived as being less than optimal but little can be done to change this situation, then cognitive dissonance reduction may act to reduce the unpleasant feelings resulting from such a housing situation (Priemus 1984, 1986; Amérigo and Aragonés 1997). The impact of future perspectives refers to the perception that one will be able to attain one’s goals sometime in the future. This means that a household may appear quite satisfied with current housing conditions, even where those conditions do not meet current needs or preferences, because of the belief that things will get better in the future (Bourne 1981). The impact of these two phenomena are explored in another study (Jansen, working paper), because there is limited scope to discuss them here.

One limitation of the current study concerns the composition of the respondent group. A sample of residents with at least a standard income was selected by a marketing bureau because of the goal of the House Buyers in Profile study, i.e., to exploring residential preferences of potential homebuyers. This does not represent the optimal group within which to explore the influence of preference and experience on housing appreciation, as the probability that these people may live in low quality housing is limited. In practice, however, not all respondents turned out to meet the income requirements and the final sample did include respondents with a lower than standard income (17 %). Furthermore, people living in relatively low quality housing (a living room of 20 m^2^, a dwelling with 2 rooms, and a backyard of 5 m length) were still represented in the current study. It would be interesting, however, to repeat this study in a group of respondents with lower than standard income.

Another limitation concerns the limited number of dwelling aspects that were examined in this study. As explained previously, this had to do with the evaluation questions being part of the larger study into residential preferences (Boumeester et al. 2008a). We had to limit ourselves to eight important dwelling aspects and could not take into consideration other important aspects of the dwelling and its environment, such as the interior and exterior of the home, relationships with neighbors, the local physical environment, and aesthetic and health features, as mentioned, for example, by Rioux and Werner (2011).

In this study, respondents were not asked specifically about their residential satisfaction. Instead, we asked about their appreciation of a range of dwelling aspects, irrespective of their own actual housing situation. Thus, the design of the current study also yielded evaluations for dwelling aspects that respondents did not currently experience. This approach may have limited the potential impact of social desirability (see, for example, Amérigo and Aragonés 1990, 1997) that might be induced by asking specifically about respondents’ satisfaction with their own actual housing situation. Furthermore, despite its limitations, this study seeks to unravel the cognitive mechanisms that influence residential satisfaction. These mechanisms are difficult to operationalize and empirical studies are therefore scarce (Amérigo 2002).

In conclusion, why is housing always satisfactory? In this paper, housing is satisfactory because the gap between what residents want and what they have is only small; residents seem to have realistic aspirations. Furthermore, residents appreciate what they already have even if this is not what they prefer.

## Appendix

### Question Concerning the Willingness to Move

Suppose that you could find a dwelling that would fulfill all your needs with regard to housing. Would you:

- Stay in your current dwelling
- or would you move?

### Question Concerning the Appreciation of the Dwelling Aspects

Now, a number of dwelling aspects will be listed, such as the number of rooms. Could you please indicate your appreciation of each dwelling aspect that is mentioned. You can indicate your appreciation by providing a number between 0 and 100. Zero means that you do not appreciate the particular dwelling aspect at all whereas 100 means that you find the particular dwelling aspect extremely attractive. Thus, a higher number is related to more attractiveness. Please take your actual housing situation and household income as a starting point in answering the questions.

Could you please indicate on a scale ranging from 0 “extremely unattractive” to 100 “extremely attractive” how you would appreciate it to live:

- in an apartment?
- etc. (see Table 2 for all dwelling aspects).

(Note that additional information was provided to explain dwelling aspects such as architectural style and residential environment).
